# Synthesis, characterization and antimicrobial properties of green-synthesised silver nanoparticles from stem bark extract of *Syzygium alternifolium* (Wt.) Walp.

**DOI:** 10.1007/s13205-015-0307-4

**Published:** 2015-05-16

**Authors:** Pulicherla Yugandhar, Reddla Haribabu, Nataru Savithramma

**Affiliations:** Department of Botany, Sri Venkateswara University, Tirupati, 517502 A.P India

**Keywords:** *Syzygium alternifolium*, Green synthesis, Silver nanoparticles, UV–Vis, FT-IR, XRD, AFM, SEM with EDAX, TEM, Antimicrobial activity

## Abstract

Today green synthesis of silver nanoparticles (SNPs) from plants is an utmost emerging filed in nanotechnology. In the present study, we have reported a green method for synthesis of SNPs from aqueous stem bark extract of *Syzygium alternifolium,* an endemic medicinal plant of South Eastern Ghats. These green-synthesised nanoparticles are characterised by colour change pattern, and the broad peak obtained at 448 nm with UV–Vis surface plasmon resonance studies confirm that the synthesised nanoparticles are SNPs. FT-IR spectroscopic studies confirm that phenols and proteins of stem bark extract is mainly responsible for capping and stabilisation of synthesised SNPs. Crystallographic studies from XRD indicates, the SNPs are crystalline in nature owing to 44 nm size. EDAX analysis shows 19.28 weight percentage of Ag metal in the sample indicates the purity of sample. AFM, SEM and TEM microscopic studies reveal that the nanoparticles are spherical in shape with sizes ranging from 4 to 48 nm. Antimicrobial studies of the synthesised SNPs on clinically isolated microbes showed very toxic effects. It indicates that stem bark extract of *S. alternifolium* is suitable for synthesising stable silver nanoparticles which act as excellent antimicrobial agents.

## Introduction

Nanotechnology is one of the most fascinating research areas in modern material science. Nanoparticles are gaining importance in the fields of biology, medicine and electronics owing to their unique physical and biological properties (Morones et al. 2005). Recent studies are focused towards synthesis of nanoparticles using plant materials like, iron, copper, calcium, gold, palladium, zinc and silver. Silver has been recognised of its importance in chemistry, physics and biology due to its unique properties. Conventional methods to synthesise silver nanoparticles are mainly by different chemical, physical and microbial approaches. The most common approach for synthesis of SNPs in chemical approach is by the use of sodium borohydride (NaBH_4_) and citrate as reducing agents. Topical exposure of NaBH_4_ severely irritates skin and eye, breathing NaBH_4_ irritates nose and throat, higher exposures can cause pulmonary edema, and very higher exposure may affect nervous system. Citrate causes hypocalcaemia, fatigue, paresthesia and muscle spasms. Common methods for the synthesis of SNPs by physical approach are laser ablation and evaporation/condensation methods. Evaporation/condensation method which could be carried out by using a tube furnace at atmospheric pressure has some disadvantages: occupies large space and consumes a great amount of power. Laser ablation method is also not a cost effective method. These chemical and physical approaches are complicated, expensive and cause potential environmental and biological hazards. In recent times, 50–120 nm-sized silver nanoparticles are synthesised from *Bacillus* species which acts as a good reducing agent (Vithiyav et al. 2014) but, a significant drawback of microbe-mediated synthesis is that it is not industrially feasible due to its lab maintenance. Therefore, the biosynthesis of SNPs using plant materials is easy, efficient and eco-friendly in comparison to chemical-mediated or microbe-mediated synthesis of SNPs (Anamika et al. 2012). Silver has long been known to have strong inhibitory and bactericidal effects as well as broad spectrum of antimicrobial activity even at low concentrations (Morones et al. 2005). Hence, among the metal nanoparticles, SNPs synthesised from medicinal plants have received much attention for their various biological properties such as anthelmintic (Seema and Amrish 2012), antilarvicidic (Sundaravadivelan et al. 2013), antioxidant (Kumara Swamy et al. 2014), anticancer (Vasanth et al. 2014), anti-inflammatory (Rafie and Hamed 2014), hepatoprotective (Bhuvaneswari et al. 2014), wound healing (Seema et al. 2014) and antimicrobial (Marutikesavakumar et al. 2014).

*S. alternifolium* belongs to the family Myrtaceae, locally known as mogi or adavineredu. It occurs in the upper plateau, slopes and valley tops with dry, slate and rocky conditions at an elevation ranging from 600 to 1000 m in Sri Venkateswara Wildlife Sanctuary of Chittoor and Cuddapah Districts of Eastern Ghats, Andhra Pradesh, India (Mohan and Lakshmi 2000). Andhra Pradesh State Biodiversity Board (Biodiversity News 2009) documented that the *S. alternifolium* is an endemic and globally endangered species as per the criteria of IUCN-CAMP to this area. Chloroform and methanolic root extract of *S. alternifolium* has analgesic and anti-inflammatory activity (Vasu et al. 2012). Leaf juice and pulp of the tender shoots are used to treat bacillary dysentery. Leaves fried in cow ghee are used as a curry to treat dry cough (Rao 2004). Some of the researchers have scientifically proven that leaves have antimicrobial activity (Raju et al. 2007), hypoglycemic and anti-hyperglycemic activity (Ramamohan et al. 2010), antioxidant (Sreelathadevi et al. 2013) and anticancer activity (Komuraiah et al. 2014). Yanadi tribe and local villagers of Veyilingalakona sacred grove and Chenchu and Nakkala tribes of Japali hanuman theertham make fruits into fine powder to treat diarrhoea (Savithramma et al. 2014a) and diabetes (Savithramma et al. 2014b), respectively. Stem bark extract used to treat gastric ulcers (Bakshu 2002), possess antiseptic properties (Rao 2004), to treat external wounds (Karuppusamy et al. 2009) and to regulate blood sugar levels (Sudhakar et al. 2012). In recent studies, synthesis of SNPs was carried out from endemic medicinal plants like *Pterocarpus santalinus* (Gopinath et al. 2013), *Boswellia ovalifoliolata* (Ankanna et al. 2010) and *Shorea tumbuggaia* (Venkateswarlu et al. 2010) which are very rich source of phenols and proteins and act as a good source towards the reduction of silver nanoparticles. There is no report on synthesis of SNPs from *S. alternifolium* stem bark so far. Hence the present study is undertaken to synthesise stable silver nanoparticles from stem bark extract of *S. alternifolium* and test their antimicrobial potentialities after characterization using advanced tools.

## Materials and methods

### Synthesis of silver nanoparticles

*S. alternifolium* stem bark was collected from Nagatheertham area of Tirumala, Chittoor District of Andhra Pradesh, India and cross checked by herbarium deposited (Voucher no. 121) in Dept. of Botany, Sri Venkateswara University, Tirupati. Finely ground 25 g of powdered stem bark was extracted with 100 ml of milli-Q water in a boiling water bath for 1 h. Filter the content with whatman no.1 filter paper and store at room temperature for green synthesis of SNPs. From this 5 ml of plant extract with 50 ml of 1 mM Ag(NO_3_)_2_ is titrated at 60–80 °C for 1 h. The contents are centrifuged at 10,000 rpm for 20 min to avoid the presence of any biological impurities. Further, it was used for characterization and antimicrobial studies.

### Characterization of silver nanoparticles

UV–Vis absorption spectrum of SNPs was measured using Spectro UV 2080 Double beam 1200 l/mm spectrophotometer. Fourier-Transform Infra Red (FT-IR) spectra of synthesised SNPs were analysed in the range of 4,000 to 500 cm^−1^ with an ALPHA interferometer (ECO-ATR), Bruker, Ettlingen, Karlsruhe, Germany by KBr pellet method. Crystalline nature of metallic silver nanoparticles was examined using an X-ray diffractometer (XRD) from Shimadzu, XRD-6000 equipped with Cu Kα radiation source using Ni as filter at a setting of 30 kV/30 mA. Atomic force microscopy (AFM) analysis was carried out by using NOVA NT-MDT SOLVER NEXT, RUSSIA. Scanning electron microscopy (SEM) and Percentage presence of silver ions in synthesised sample was done by using a FEI Quanta 200 FEG HR-SEM machine equipped with EDAX instrument. Transmission electron microscopy (TEM) analysis was performed by using HF-3300 advanced 300 kV TEM/STEM from Hitachi.

### Antimicrobial studies of silver nanoparticles

The extracts of synthesised SNPs from stem bark of *S. alternifolium* was analysed for antimicrobial activity against two gram positive bacterial strains like *Bacillus subtilis*, *Staphylococcus aureus* and five Gram negative bacterial strains like *Escherichia coli*, *Klebsiella pneumoniae*, *Proteus vulgaris*, *Pseudomonas aeruginosa* and *Salmonella typhimurium*. Antifungal studies were also carried out on selective five fungal strains like *Alternaria solani*, *Aspergillus niger*, *Aspergillus flavus*, *Penicillium chrysogenum* and *Trichoderma harzianum* procured from Dept. of Microbiology, Sri Venkateswara University, Tirupati. Disc diffusion assay method was followed using standard protocol (Cruickshank 1986). For this 20 µl of 50 µg/ml concentration of plant extract, synthesised SNPs, streptomycin/fluconazole and 1 mM concentration of Ag(NO_3_)_2_ solution are applied on separate filter paper discs (Whatman No. 1 filter paper with 7 mm diameter) and allowed to dry before being placed on the agar medium. Triplicates of each extract was tested and incubated at 37  °C for 24 h. in incubation chamber. Diameter of the zones was measured in centimetres (cm) with the help of scale and the results were tabulated.

## Results and discussion

**Fig. 1:**
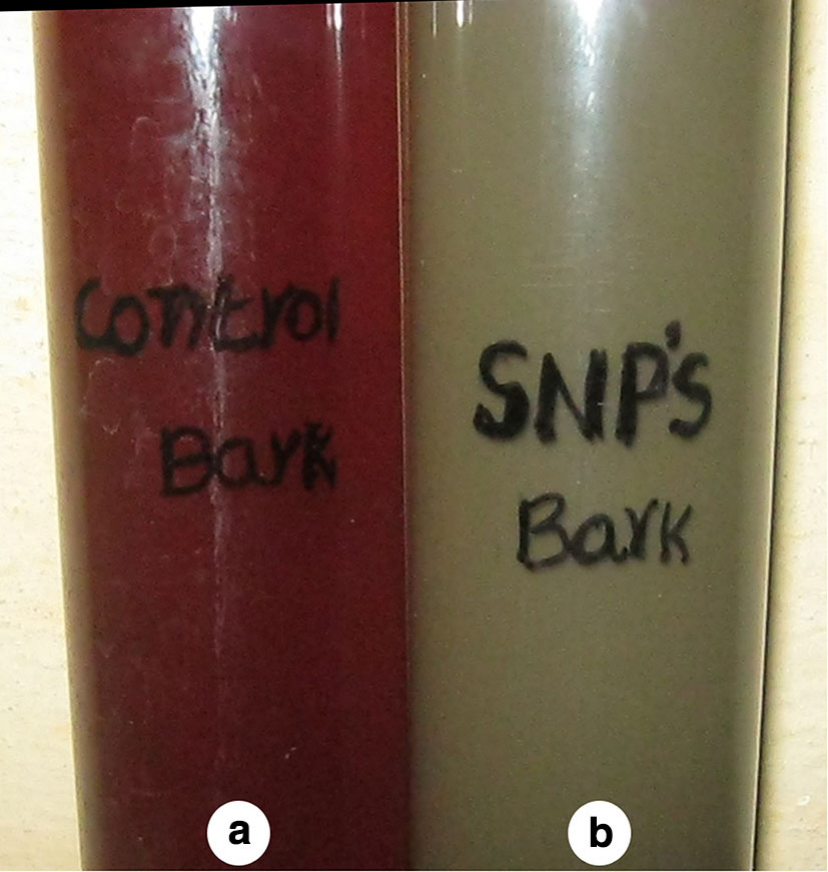
Colour change pattern of synthesised SNPs: **a** *brown*, **b** *grey*

**Fig. 2:**
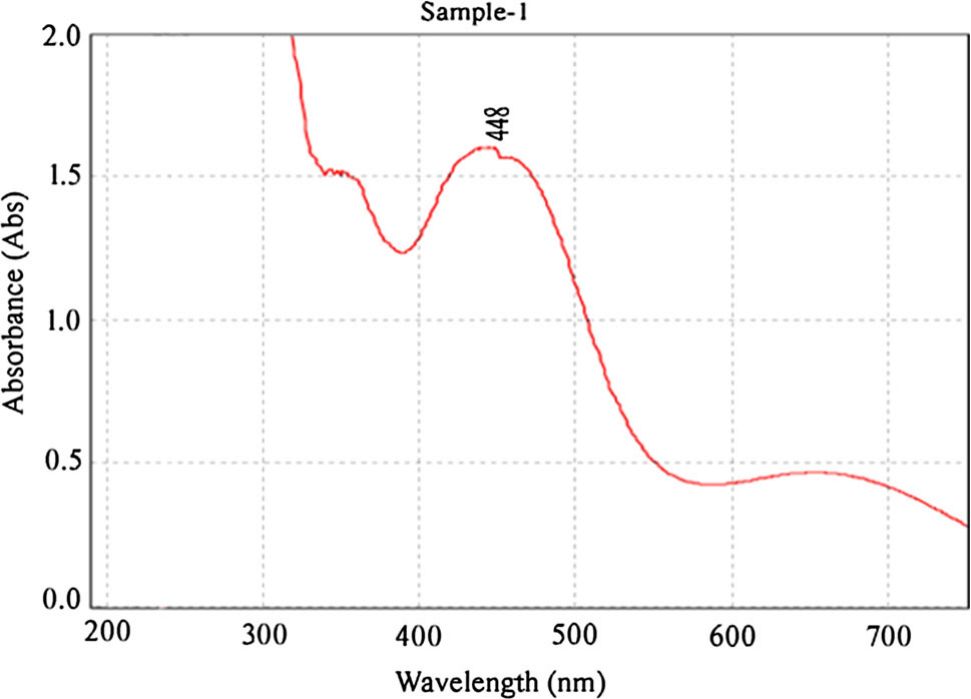
Surface plasmon resonance analysis of synthesised SNPs with UV–Vis spectroscopy shows a typical broad peak at 448 nm

**Fig. 3:**
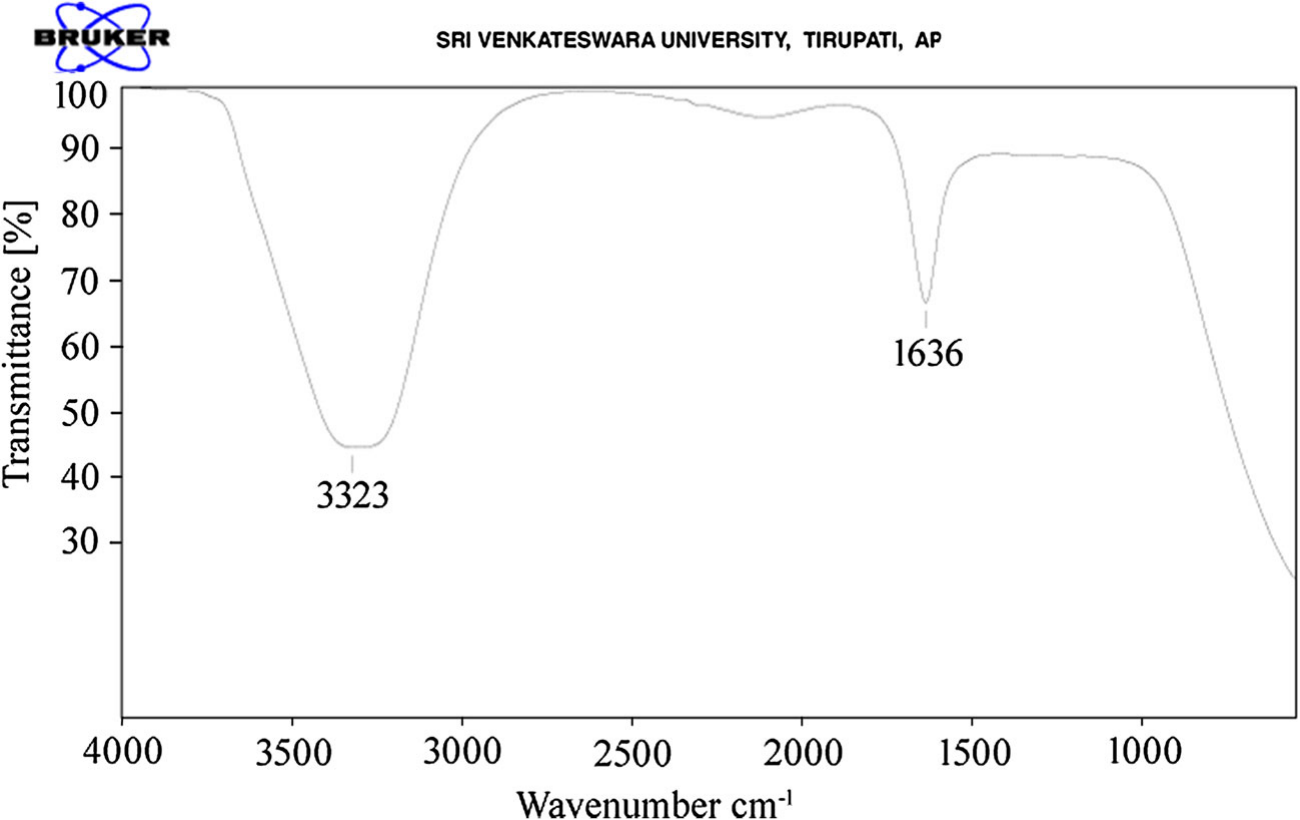
FT-IR analysis of synthesised SNPs shows broad peaks at 3323 cm^−1^ of phenols and 1636 cm^−1^ of primary amines of proteins

When the aqueous stem bark extract of *S. alternifolium* was mixed with 1 mM Ag(NO_3_)_2_ solution, the colour changed from brown to grey which is the primary method to confirm that the synthesised nanoparticles are silver (Fig. 1). The colour change is due to the reduction of silver ions with the help of bio molecules present in the sample (Sankar et al. 2014). NAD and ascorbic acid present at higher levels in all plant parts act as strong reducing agents by donating electrons to Ag^+^ ions and reduced to form Ag^0^ nanoparticles (Ahmad et al. 2011). This may be the main reason behind the reduction and colour change pattern of SNPs. Reduction of these silver ions was monitored by using UV–Vis spectroscopy from 190 to 750 nm scan range. The peak obtained at 448 nm is a typical absorption peak for metallic nanoparticles which further confirms the reduced nanoparticles are silver (Fig. 2). Same type of results was found in leaf-mediated synthesis of silver nanoparticles from *Albizia adianthifolia* (Gengan et al. 2013). Here, the nanoparticles in reaction mixture absorb light at different wavelengths and get excited due to charge density at the interface between conductor and insulator of UV–Vis spectroscope to give a respective peak. This mechanism is known as surface plasmon resonance (SPR). FT-IR spectrum of synthesised SNPs was carried out to know the possible bio-molecules responsible for the capping and stabilisation of nanoparticles. For this, the sample was analysed in the scan range from 4000 to 500 cm^−1^ of near IR spectra by FT-IR. The broad peaks obtained at 3323 cm^−1^ and 1636 cm^−1^ were assigned for O–H bond of phenols and N–H bond of primary amines, respectively(Fig. 3). This suggests that the hydroxyl groups of phenols and amide groups of proteins forming a layer of the nanoparticles, act as capping agents to prevent agglomeration and provide stability to the reaction medium. Same type of results was found in *Myristica fragrans* seed extract-mediated synthesis of silver nanoparticles (Sharma et al. 2014).

**Fig. 4:**
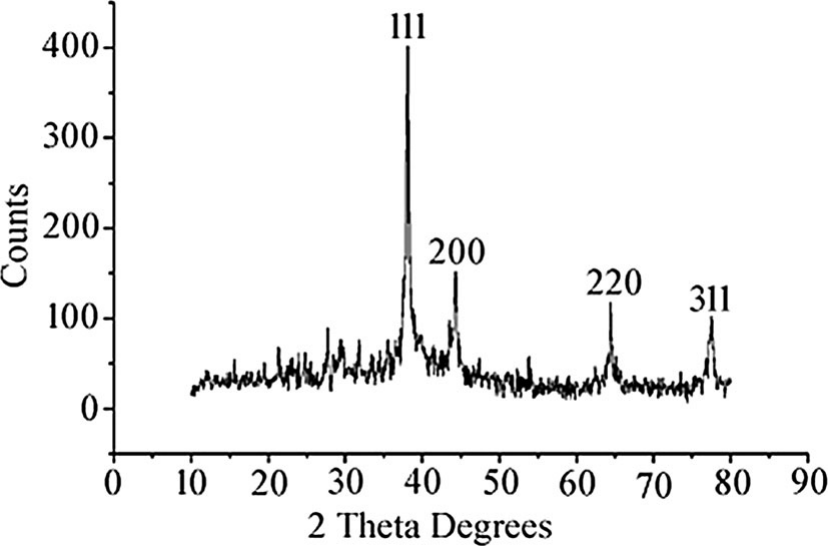
XRD crystallographic studies of synthesised SNPs show four intensive peaks with 44 nm size

**Fig. 5:**
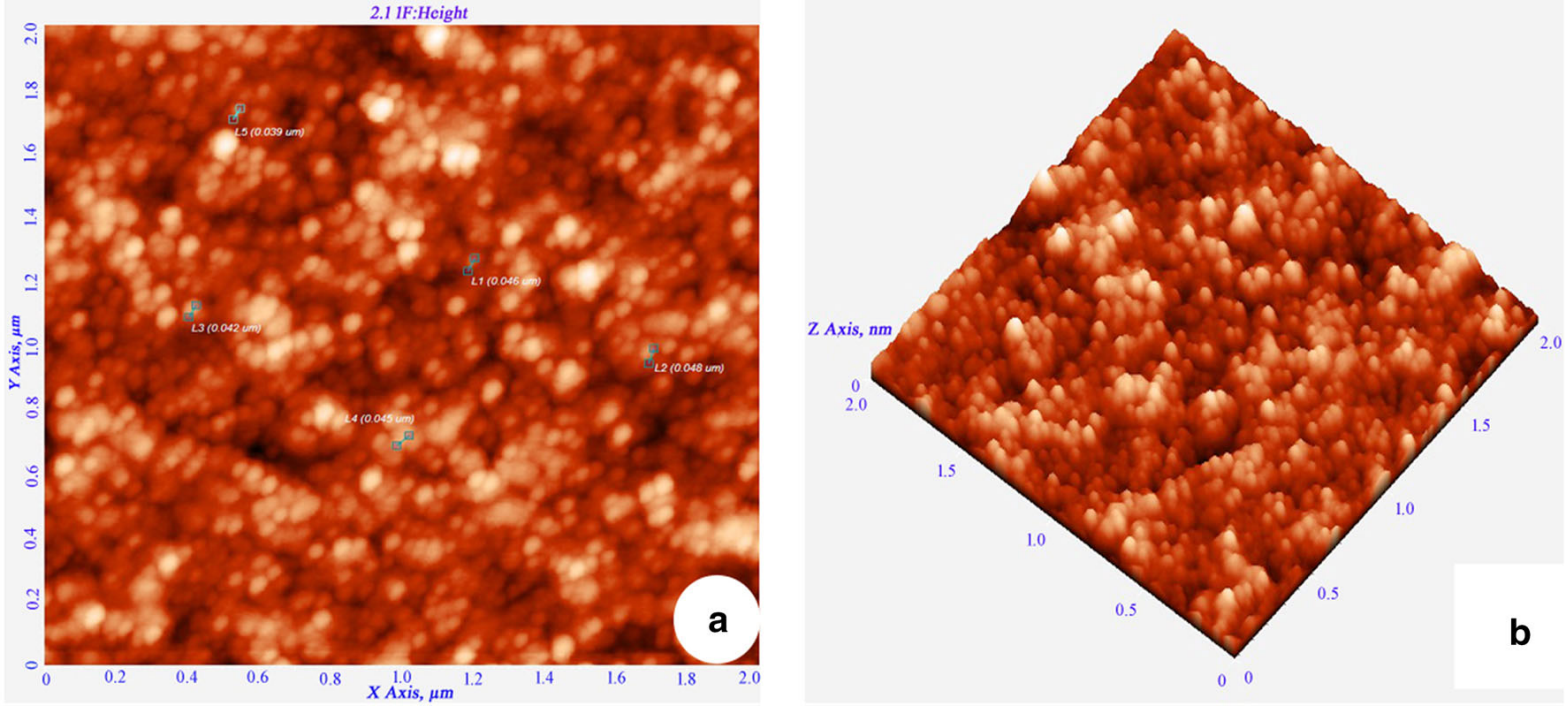
**a** 2 × 2 µm resolution studies of synthesised SNPs with AFM analysis show size range from 34 to 49 nm, spherical shape and without any agglomeration of particles, **b** 3D micrograph of synthesised SNPs

**Fig. 6:**
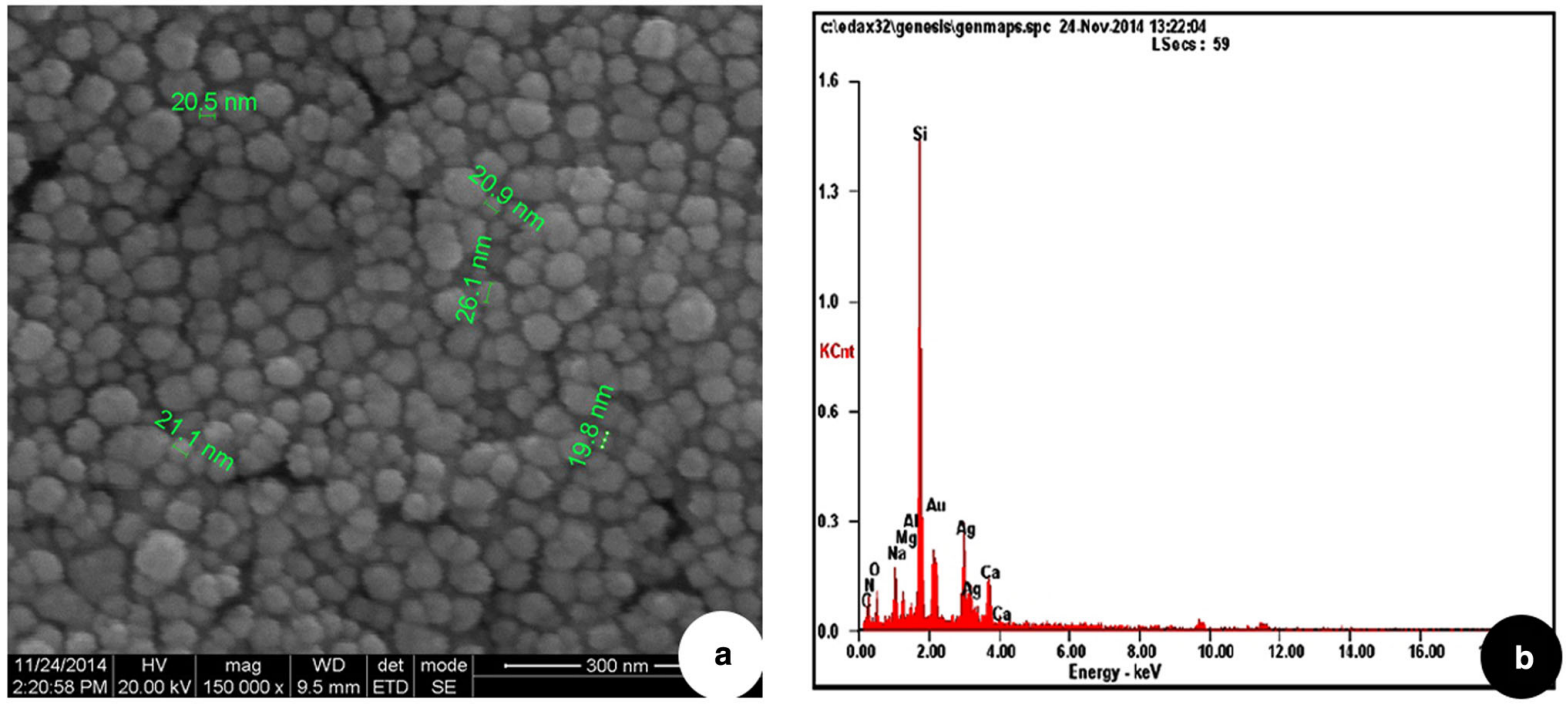
**a** 300 nm resolution studies of synthesised SNPs with SEM analysis show size range from 19 to 26 nm with spherical-shaped particles, **b** EDAX analysis of synthesised SNPs shows 19.28 weight percentage of Ag metal

**Fig. 7:**
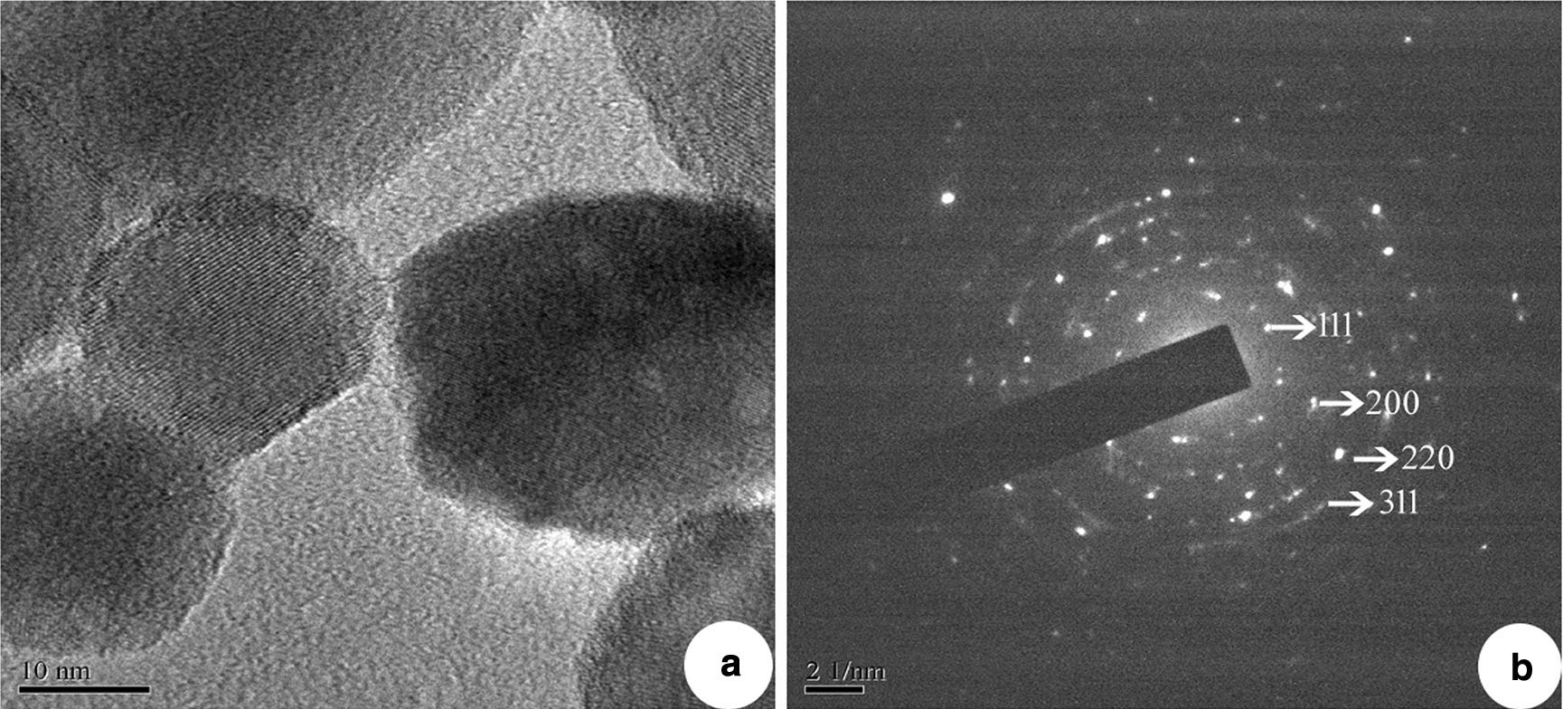
**a** 10 nm resolution studies of synthesised SNPs with TEM analysis show size range from 5 to 10 nm, spherical shaped and without any agglomerated particles, **b** SAED pattern of TEM analysis of synthesised SNPs shows crystallographic nature of nanoparticles

X-ray diffraction analysis was carried out to confirm the nature of nanoparticles. The XRD pattern shows four peaks on 2θ° of *X*-axis like 38.40°, 46.20°, 64.50° and 77.30° corresponding to 111, 200, 220 and 311 Bragg reflections of *Y*-axis, respectively (Fig. 4). These Bragg reflections confirm the face-centred cubic structure of SNPs and it coincides with powder diffraction file of International Centre for Diffraction Data (No. 04-07830) and has 44 nm size of SNPs calculated according to Debye–Scherrer equation ((*D* = *kλ*/*β* cos θ). AFM was used as a primary method to monitor the dissolution and agglomeration pattern of surface topology of nanoparticles. The 2 µm resolution studies of AFM reveal that the particles are spherical in shape, with size ranging from 39 to 48 nm (Fig. 5a). Raw data obtained from AFM microscope are treated with specially designed image processing software (NOVA-TX) to further exploit the 3D image of the nanoparticles (Fig. 5b). 500 nm resolution studies of synthesised SNPs with SEM analysis show that the particles are polydispersed, with size ranging from 19 to 26 nm, owing to spherical shape without any agglomeration (Fig. 6a). The EDAX analysis of synthesised sample shows 19.28 weight percentage of Ag metal along with 15.18 % of carbon, 03.64 % of nitrogen, 07.58 % of oxygen, 04.24 % of sodium, 01.75 %of magnesium, 0.70 % of aluminium, 24.60 % of silicon, 18.15 % of aurum and 04.87 % of calcium. 19.28 % of silver indicates the sample having high purity of silver nanoparticles (Table 1; Fig. 6b). A higher magnification study was carried out with TEM analysis to know the size and shape of the nanoparticles along with crystalline nature. 10 nm resolution studies of synthesised SNPs show 4–7 nm size with spherical shape and they do not have any agglomeration (Fig. 7a). Crystallographic nature of SNPs by selected area electron diffraction (SAED) pattern shows fringe array of spots corresponding to 111, 200, 220 and 311 (Fig. 7b). The Bragg reflections of XRD pattern is correlated with this SAED pattern of SNPs, which clearly indicates that the nanoparticles are crystalline in nature with face-centred cubic structures. AFM, SEM and TEM microscopic studies of green-synthesised nanoparticles from *S. alternifolium* show polydispersed 4–48 nm sized spherical-shaped particles.

**Table 1:** EDAX analysis of synthesised SNPs shows 19.28 weight percentage of Ag metal

S. no	Element	Weight (%)
1.	C	15.18
2.	N	03.64
3.	O	07.58
4.	Na	04.24
5.	Mg	01.75
6.	Al	00.70
7.	Si	24.60
8.	Au	18.15
9.	Ag	19.28
10.	Ca	04.87

**Fig. 8:**
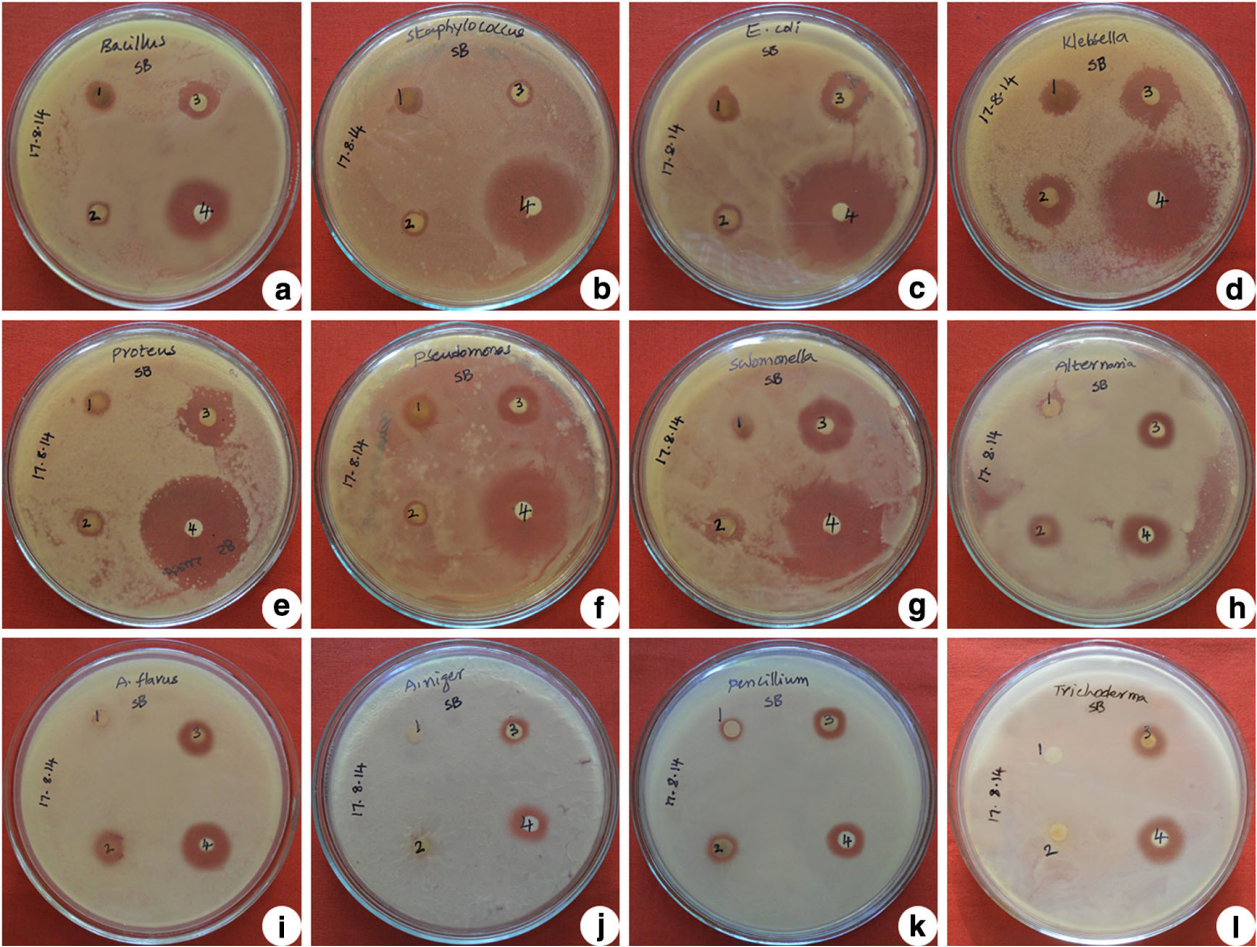
Antimicrobial activity of green synthesised silver nanoparticles from stem bark extract of *S. alternifolium*: **a** *B. subtilis*, **b** *S. aureus*, **c** *E. coli*, **d** *K. pneumoniae*, **e** *P. vulgaris*, **f** *P. aeruginosa*, **g** *S. typhimurium*, **h** *A. solani*, **i** *A. flavus*, **j** *A. niger*, **k** *P. chrysogenum and* **l** *T. harzianum*; *1* Plant extract, *2* Ag (NO_3_) _2_, *3* SNPs, *4* streptomycin/fluconazole

**Fig. 9:**
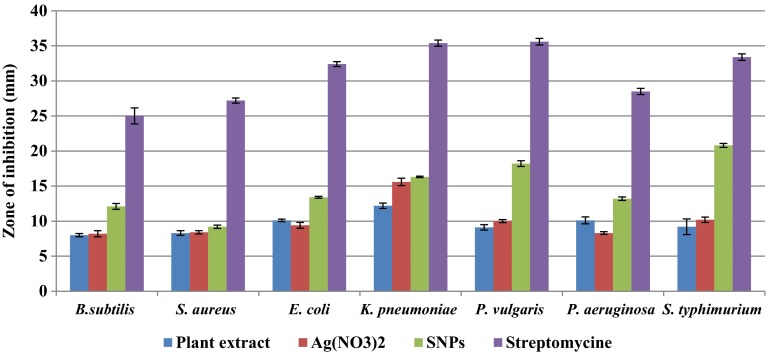
Zone of inhibition of different extracts on clinically isolated bacteria

**Fig. 10:**
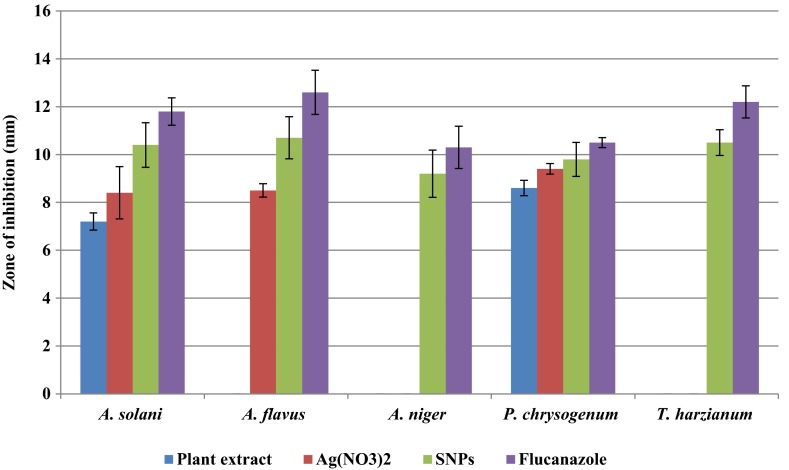
Zone of inhibition of different extracts on clinically isolated fungi

Antimicrobial activity of green synthesised SNPs are analysed on two gram negative and five gram positive bacterial strains growing on nutrient agar medium and five fungal strains growing on potato dextrose agar medium. The inhibition zone of each extract is compared with standard drugs like streptomycin for bacteria and fluconazole for fungi. These SNPs show highest inhibitory activity on *S. typhimurium* followed by *P. vulgaris*, *K. pneumoniae*, *E. coli*, *P. aeruginosa*, *S. aureus* and *B. subtilis* (Figs. 8; Table 2; Fig. 9). Whereas in the case of fungi, highest inhibition zones are observed in *A. flavus* followed by *P. chrysogenum*, *T. harzianum*, *A. solani* and *A. niger* (Figs. 8; Table 3; Fig. 10). Based on these studies 4–48 nm size spherical-shaped nanoparticles show higher toxicity towards clinically isolated microorganisms when compared with Ag(NO_3_)_2_ solution as negative control. Spherical-shaped SNPs, 20–60 nm in size, synthesised from stem bark of *Syzygium cumini* show good antibacterial activity (Prasad and Swamy 2013). Spherical shaped SNPs, 20–35 nm in size, synthesised from *Cochlospermum religiosum* stem bark show significant antimicrobial activity on different bacterial and fungal pathogens (Sasikala et al. 2014). In our study, gram negative bacteria shows more susceptibility towards SNPs when compared to Gram positive bacteria. The Gram positive bacteria having thick layers of peptidoglycans (together with polypeptide contains proteins) and penetration of SNPs through cell membrane are not that much of easy when compared to gram negative bacteria. The results revealed that the bacterial strains show higher susceptibility to SNPs when compared to fungal strains. Because the fungal cell walls are made up of chitin (a protein polymer) having parallel strands of glucosamines linked with β, 1–4 linkages and cross-linked with hydrogen bonds. Whereas in the case of bacteria, the cell walls are made up of peptidoglycan (a carbohydrate polymer) linked by amino acids and cross-linked by tetrapeptides. Hence the bacterial cell walls are more flexible and elastic, whereas fungal cell walls are somewhat rigid than the bacterial cell walls. Due to this, penetration of SNPs through rigid fungal cell walls is somewhat difficult when compared to bacterial cell walls. Silver is a precious metal used as an effective antimicrobial agent before the advent of silver nanoparticles. The overuse of silver agents has decreased the efficiency of silver agents as an antibiotic. In the recent times, the advancement of nanotechnology has rekindled the interest in the use of silver nanoparticles as antibacterial agents (Wong and Liu 2010). SNPs may attach to the cell membrane surface of pathogens, disturb their permeability and cause structural changes in bacteria (Sondi and Sondi 2004) and destruct the membrane integrity in fungal spores (Krishnaraj et al. 2012), eventually leading to cell death. Some scientists state that the SNPs penetrate inside the bacteria and fungi and cause damage by interacting with the electron phosphorous and sulphur containing compounds such as DNA and proteins, resulting in cell death (Baker et al. 2005). Small size, spherical shape and high surface area to volume ratio to interact with cell walls of pathogens gives them better antimicrobial activity (Agnihotri et al. 2014). The present study also clearly reveals that the particle size ranging from 4 to 48 nm with spherical shape proves high potential microbial activity.

**Table 2:** Comparison of different extracts of green synthesised silver nanoparticles effect on different clinically isolated bacteria

S. no	Name of the organism	Plant extract	Ag(NO_3_)_2_	SNPs	Streptomycin
1.	*B.* *subtilis*	8.0 ± 0.24	8.2 ± 0.43	12.1 ± 0.41	25.0 ± 1.15
2.	*S. aureus*	8.3 ± 0.33	8.4 ± 0.24	9.2 ± 0.24	27.2 ± 0.37
3.	*E. coli*	10.1 ± 0.18	9.4 ± 0.40	13.4 ± 0.14	32.4 ± 0.34
4.	*K. pneumoniae*	12.2 ± 0.38	15.6 ± 0.53	16.3 ± 0.10	35.4 ± 0.44
5.	*P. vulgaris*	9.1 ± 0.38	10.0 ± 0.22	18.2 ± 0.42	35.6 ± 0.47
6.	*P. aeruginosa*	10.1 ± 0.50	8.3 ± 0.20	13.2 ± 0.24	28.5 ± 0.44
7.	*S. typhimurium*	9.2 ± 1.11	10.2 ± 0.38	20.8 ± 0.28	33.4 ± 0.46

Values are average of triplicates

±, SE

**Table 3:** Comparison of different extracts of green-synthesised silver nanoparticles effect on different clinically isolated fungi

S. no	Name of the organism	Plant extract	Ag(NO_3_)_2_	SNPs	Fluconazole
1.	*A. solani*	7.2 ± 0.36	8.4 ± 1.09	10.4 ± 0.93	11.8 ± 0.57
2.	*A. flavus*	0	8.5 ± 0.28	10.7 ± 0.88	12.6 ± 0.92
3.	*A. niger*	0	0	9.2 ± 0.99	10.3 ± 0.88
4.	*P. chrysogenum*	8.6 ± 0.32	9.4 ± 0.22	9.8 ± 0.71	10.5 ± 0.21
5.	*T. harzianum*	0	0	10.5 ± 0.54	12.2 ± 0.67

Values are average of triplicates

0, no inhibition zone; ±, SE

## Conclusion

The present study is aimed to develop a fast, eco-friendly and cost effective method for the synthesis of silver nanoparticles from *S. alternifolium*. Due to significant drawbacks with physical, chemical- and microbe-mediated methods of silver nanoparticles, green synthesis is the best method. These green-synthesised SNPs are polydispersed, without any agglomeration and have sizes ranging from 4 to 48 nm with spherical shape show broad spectrum of antimicrobial activity against different clinically isolated bacteria and fungi by acting as a potential antimicrobial agent. High amount of small-sized nanoparticles produced with little amount of plant extract is beneficial because it is an endemic and endangered medicinal plant. Based on these results, we conclude that *S. alternifolium* stem bark is an efficient and effective source for the synthesis of silver nanoparticles.
